# The Use of Metabolomics and Inflammatory Mediator Profiling Provides a Novel Approach to Identifying Pediatric Appendicitis in the Emergency Department

**DOI:** 10.1038/s41598-018-22338-1

**Published:** 2018-03-06

**Authors:** Nusrat S. Shommu, Craig N. Jenne, Jaime Blackwood, Dori-Ann Martin, Ari R. Joffe, Robin Eccles, Mary Brindle, Ijab Khanafer, Hans J. Vogel, Graham C. Thompson

**Affiliations:** 1 Bio-NMR Center, Department of Biological Sciences, University of Calgary, Calgary, AB Canada; 2 Department of Microbiology, Immunology and Infectious Diseases, University of Calgary, Calgary, AB Canada; 3 Department of Pediatrics, University of Calgary, Calgary, AB Canada; 4 Division of Pediatric Critical Care, University of Alberta, Edmonton, AB Canada; 5 Department of Surgery, University of Calgary, Calgary, AB Canada; 6 Department of Emergency Medicine, University of Calgary, Calgary, AB Canada

## Abstract

Multiplexed profiling approaches including various ‘omics’ platforms are becoming a new standard of biomarker development for disease diagnosis and prognosis. The present study applied an integrated metabolomics and cytokine profiling approach as a potential aid to the identification of pediatric appendicitis. Metabolic analysis using serum (n = 121) and urine (n = 102) samples, and cytokine analysis using plasma (n = 121) samples from children presenting to the Emergency Department with abdominal pain were performed. Comparisons between children with appendicitis vs. non-appendicitis abdominal pain, and with perforated vs. non-perforated appendicitis were made using multivariate statistics. Serum and urine biomarker patterns were statistically significantly different between groups. The combined serum metabolomics and inflammatory mediator model revealed clear separation between appendicitis and non-appendicitis abdominal pain (AUROC: 0.92 ± 0.03) as well as for perforated and non-perforated appendicitis (AUROC: 0.88 ± 0.05). Urine metabolic analysis also demonstrated distinction between the groups appendicitis and non-appendicitis abdominal pain (AUROC: 0.85 ± 0.04), and perforated and non-perforated appendicitis (AUROC: 0.98 ± 0.02). In children presenting to the Emergency Department with abdominal pain, metabolomics and inflammatory mediator profiling are capable of distinguishing children with appendicitis from those without. The approach also differentiates between severities of disease. These results provide an important first step towards a potential aid for improving appendicitis identification.

## Introduction

Acute appendicitis is the most common non-traumatic presentation to the pediatric emergency department (PED) that requires surgical consultation^1,2^. At times, accurate diagnosis of appendicitis is quite challenging in pediatric patients, as they may not demonstrate the typical signs and symptoms^3–6^ and other conditions can also produce abdominal pain that mimics acute appendicitis in children^3,4^.

Biomarkers indicating the presence of infection (white blood cell count, neutrophil count) and subsequent recruitment of the immune/inflammatory and metabolite cascades (c-reactive protein, procalcitonin, 5-hydroxyindoleacetic acid) are often used by clinicians to assist diagnosis^3,7^. However, to date, no single bio-marker has demonstrated sufficient predictive power in pediatric appendicitis. Moreover, imaging studies (ultrasounds, computed tomography, magnetic resonance imaging) that are often used for the diagnosis of pediatric appendicitis may be limited by factors related to accuracy, availability, or radiation exposure^8,9^. Indeed, significant practice variation in the diagnosis of pediatric appendicitis has been documented^10^, further illustrating the need for innovative alternative detection methods.

In the present study we aim to evaluate the potential of a novel integrated metabolomics- and inflammatory mediators- based biomarker development approach to aid the diagnosis of acute appendicitis in children. Metabolites represent the intermediary and final products of the metabolic pathways within an organism; therefore, it is possible to achieve unique insight by studying these compounds under any given physiological condition^11^. In addition, profiling of the extended spectrum of inflammatory mediators could provide an expanded understanding of the overall immune response. In a previous pilot study we demonstrated the feasibility of this innovative approach for differentiating pediatric appendicitis patients from pediatric control patients with no abdominal pain (manuscript submitted). The aim of the present study is to apply this approach to accurately distinguish children presenting to the PED with abdominal pain as (a) pathology proven appendicitis from negative pathology and (b) non-perforated appendicitis from perforated appendicitis.

## Results

### Sample description

**Figure 1:**
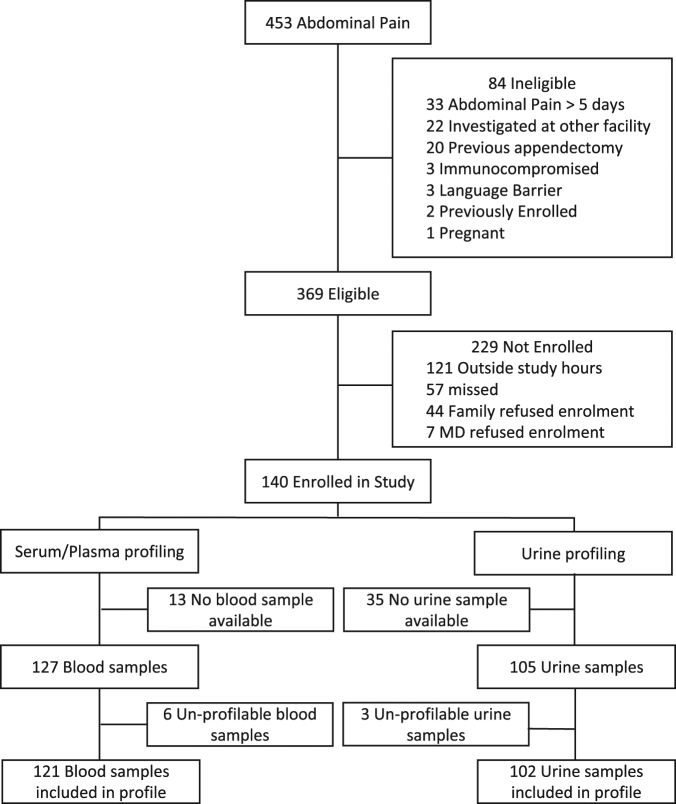
Screening, enrolment and analysis of children with suspected appendicitis.

Blood samples from 121 pediatric patients (76 non-appendicitis ‘NA’, 32 non-perforated appendicitis ‘NPA’, and 13 perforated appendicitis ‘PA’) and urine samples from 102 patients (66 ‘NA’, 27 ‘NPA’, and 9 ‘PA’) were available for analysis. Figure 1 outlines patient enrolment and inclusion; demographic and clinical characteristics of the participants are portrayed in Table 1. The demographics and clinical features of children based on pathology classification are described in the supplementary Table S1. A total of 102 metabolites and 54 inflammatory compounds from serum, and 222 metabolites from urine could be reliably detected from the patients. A comprehensive list of the metabolites detected is available from the authors upon reasonable request.

**Table 1:** Demographics, Clinical Features and Investigations of children with abdominal pain and suspected appendicitis.

	Enrolled (n = 140)	Blood Profile (n = 121)	Urine Profile (n = 102)
Age, mean (SD)	11.6 (3.4)	11.7 (3.4)	11.6 (3.3)
Male, n (%)	61 (43.6)	55 (45.5)	43 (42.2)
Prior health care visit for same illness, n (%)	61 (43.6)	56 (46.3)	43 (42.2)
Nausea, n (%)	95 (67.9)	80 (66.1)	71 (69.6)
Vomiting, n (%)	68 (48.6)	57 (47.1)	52 (51.0)
Anorexia/poor appetite, n (%)	101 (72.1)	85 (70.3)	73 (71.6)
Fever in the ED (>38 C), n (%)	37 (26.4)	34 (28.1)	27 (26.5)
RLQ pain, n(%)	106 (75.7)	93 (76.9)	81 (79.4)
Pediatric Appendicitis Score, median (IQR)	6 (4)	6 (4)	6 (4)
WBC, mean (SD)	11.8 (5.0)	11.8 (5.1)	11.7 (5.1)
Neutrophils, mean (SD)	8.9 (5.1)	8.8 (5.2)	8.8 (5.2)
Ultrasound completed, n (%)	136 (97.1)	118 (97.5)	99 (97.1)
CT completed, n (%)	10 (7.1)	9 (7.4)	10 (9.8)
Surgical Consult in the ED, n (%)	96 (68.6)	81 (66.9)	70 (68.6)
ED disposition			
Discharged home, n (%)	66 (47.1)	57 (47.1)	48 (47.1)
Admit to OR, n (%)	55 (39.3)	48 (39.7)	36 (35.3)
Admit to surgical ward for observation, n (%)	16 (11.4)	13 (10.7)	15 (14.7)
Admit to medical ward, n (%)	3 (2.1)	3 (2.5)	3 (2.9)
Appendectomy, n (%)	57 (40.7)	50 (41.3)	38 (37.3)
Appendicitis, n (%)	52 (37.1)	47 (38.8)	36 (35.3)
Negative Appendectomy Rate	8.8%	6.0%	5.3%

Abbreviations: ED – Emergency Department; RLQ – Right Lower Quadrant; WBC – White Blood Cell count; CT – Computed Tomography; MRI – Magnetic Resonance Imaging.

### Serum metabolic and cytokine profiling

**Figure 2:**
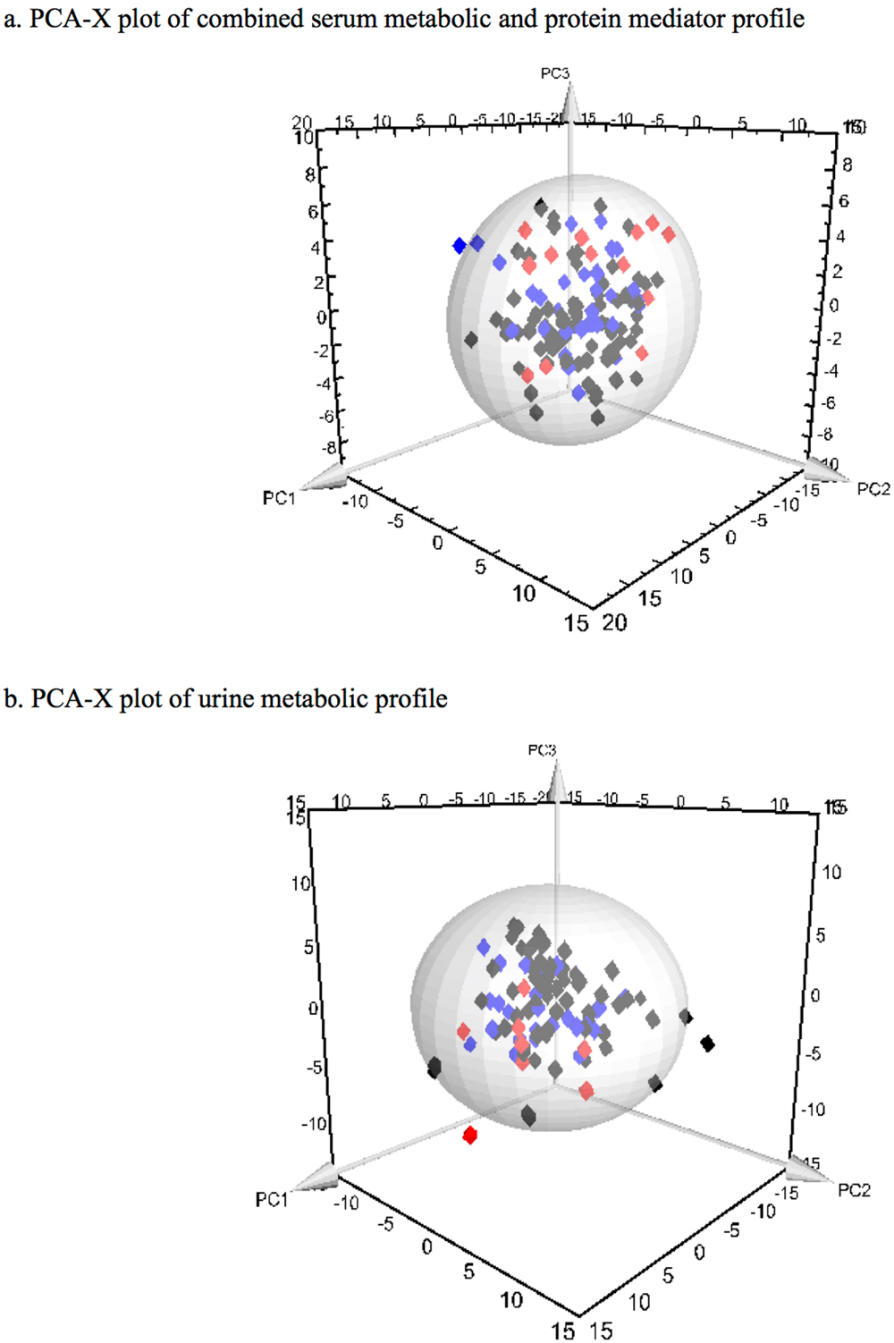
Three-dimensional PCA-X score scatter plots of pediatric patients with and without appendicitis using **(a)** serum metabolomic and inflammatory mediator profiling, and **(b)** urine metabolomic profiling. Each plot is based on three principal components-PC1, PC2 and PC3. Red diamonds represent distribution of pediatric patients with perforated appendicitis, blue diamonds represent patients with non-perforated appendicitis, and black diamonds represent pediatric patients with non-appendicitis abdominal pain.

**Figure 3:**
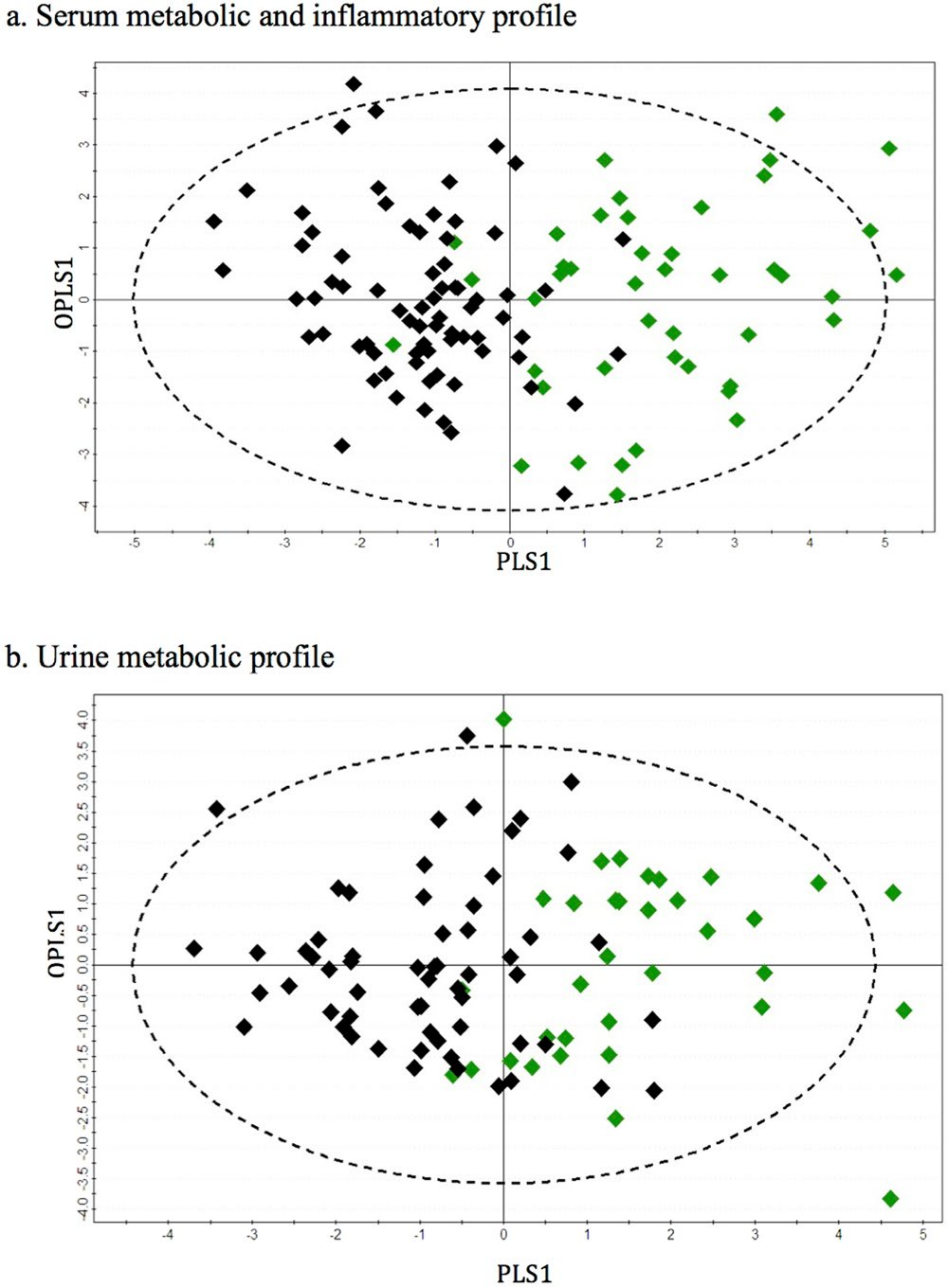
OPLS-DA score scatter plots distinguishing pediatric patients with abdominal pain, positively and negatively diagnosed with appendicitis using **(a)** serum metabolomic and inflammatory mediator profiling (R^2^Y = 0.60, Q^2^ = 0.48), and **(b)** urine metabolomic profiling (R^2^Y = 0.47, Q^2^ = 0.32). Green diamonds represent distribution of pediatric patients with appendicitis abdominal pain and black diamonds represent pediatric patients with non-appendicitis abdominal pain.

**Figure 4:**
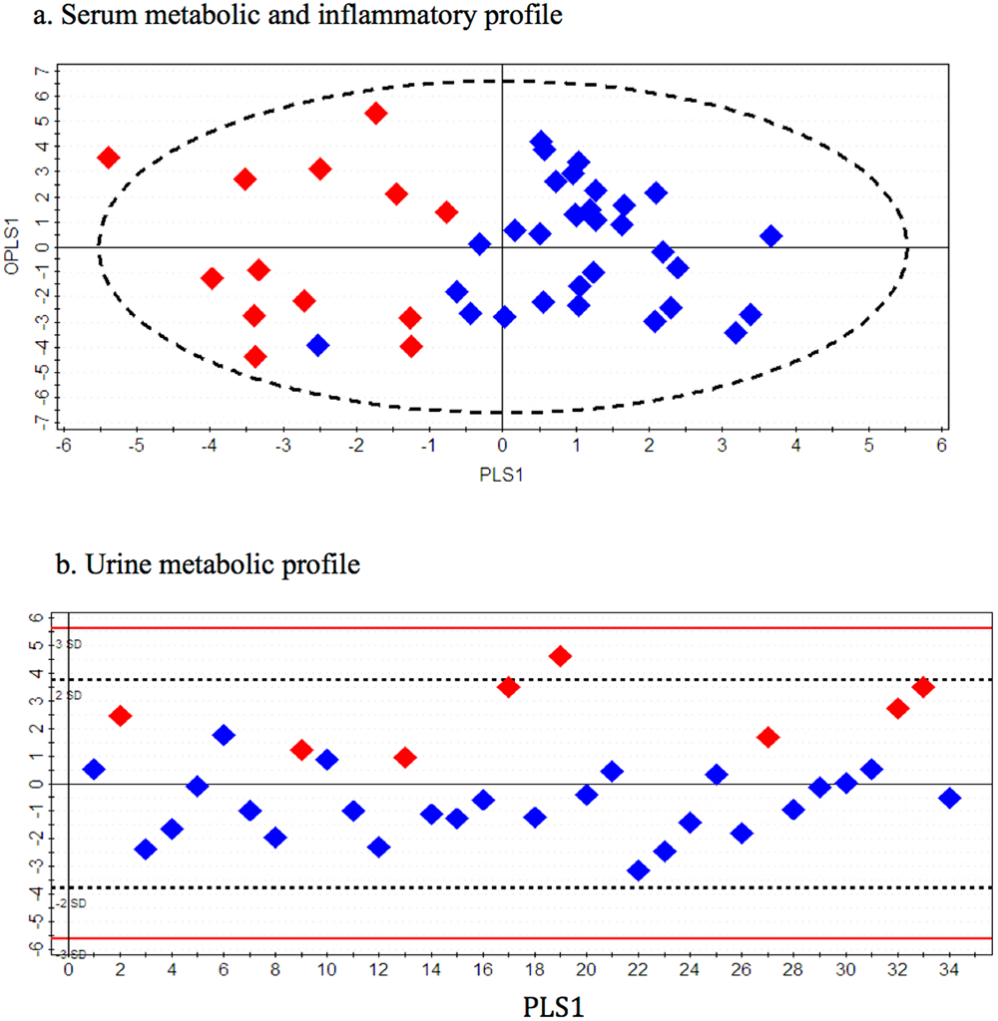
OPLS-DA score scatter plots distinguishing pediatric patients with perforated and non-perforated appendicitis using **(a)** serum metabolomic and inflammatory mediator profiling (R^2^Y = 0.66, Q^2^ = 0.37), and **(b)** urine metabolomic profiling (R^2^Y = 0.60, Q^2^ = 0.52). Red diamonds represent distribution of pediatric patients with perforated appendicitis and blue diamonds represent pediatric patients with non-perforated appendicitis.

**Figure 5:**
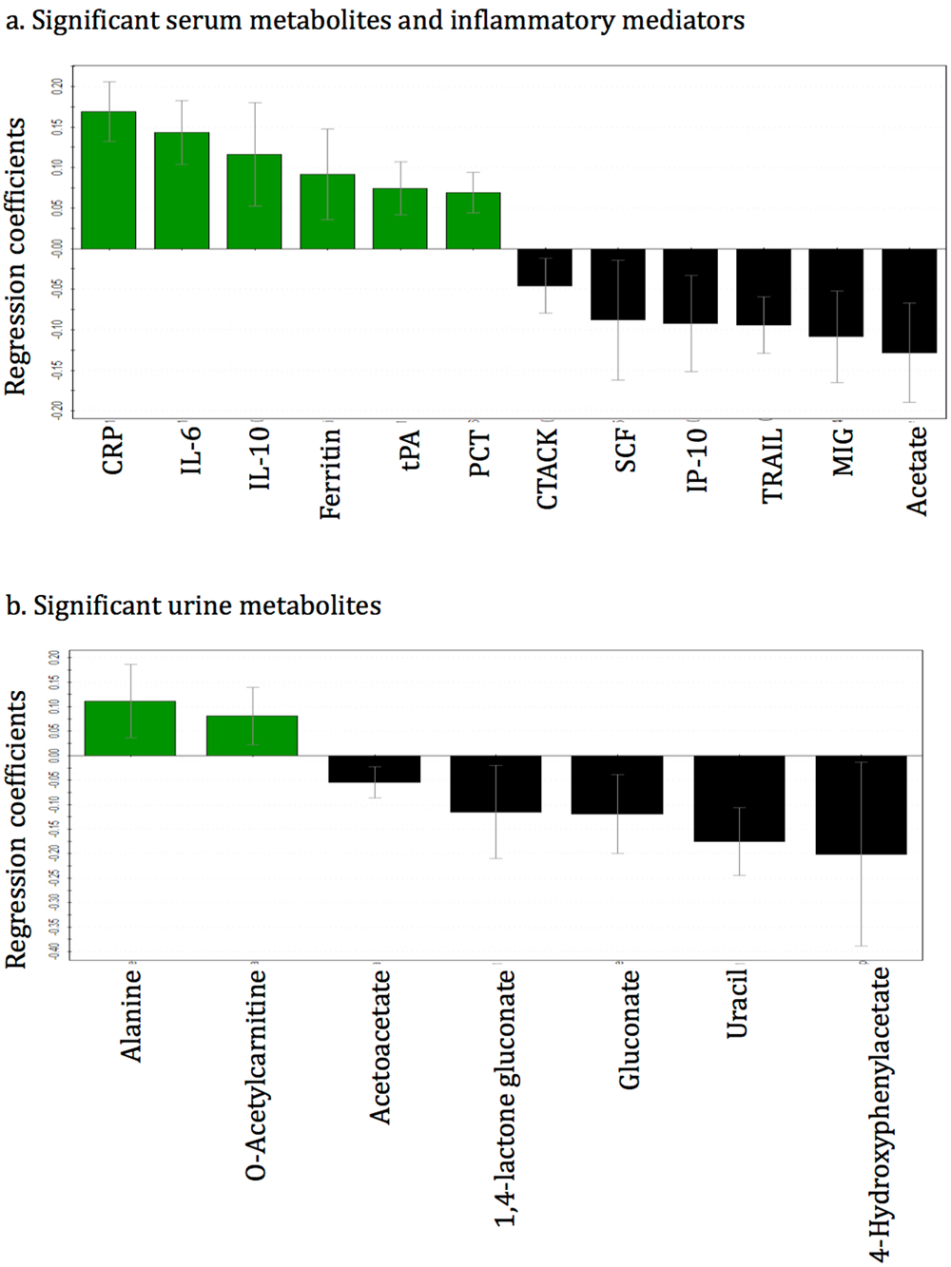
The regression coefficient plots for the statistically significant (p < 0.05) **(a)** serum metabolites and inflammatory mediators and **(b)** urine metabolites distinguishing children with appendicitis abdominal pain and non-appendicitis abdominal pain. Green bars with positive coefficient values represent increased concentrations in the appendicitis children, and black bars with negative values represent reduced concentration in the appendicitis patients, compared with non-appendicitis children. Abbreviations: CRP-C-reactive protein, IL-Interleukin, tPA-Tissue plasminogen activator, PCT-Procalcitonin, CTACK-Cutaneous T-cell-attracting chemokine, SCF-Stem cell factor, IP-10-Interferon gamma-induced protein 10, TRAIL-Tumor necrosis factor-related apoptosis-inducing ligand, and MIG-Monokine induced by gamma interferon.

**Figure 6:**
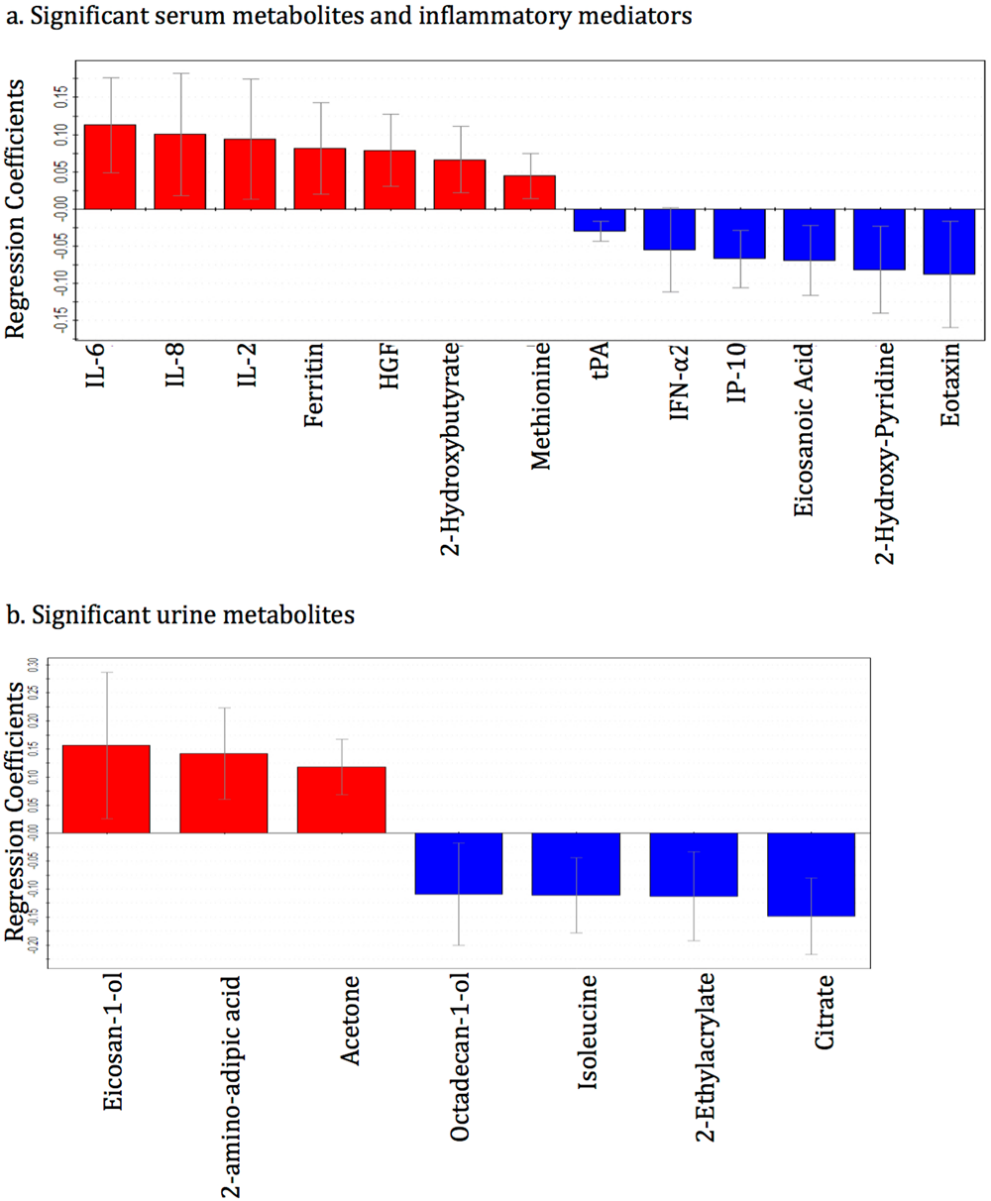
The regression coefficient plots for the statistically significant (p < 0.05) **(a)** serum metabolites and inflammatory mediators and **(b)** urine metabolites distinguishing children with perforated appendicitis and non-perforated appendicitis. Red bars with positive coefficient values represent increased concentrations in the perforated appendicitis children, and blue bars with negative values represent reduced concentration in the perforated appendicitis patients, compared with non-perforated appendicitis children. Abbreviations: IL-Interleukin, HGF-Hepatocyte growth factor, tPA-Tissue plasminogen activator, IFN-α2-Interferon alpha-2, and IP-10-Interferon gamma-induced protein 10.

Three principal components (PC1, PC2 and PC3) were calculated to build the unsupervised PCA model for the serum metabolite and cytokine combined dataset, which contributed 21.6%, 6.9% and 4.7% percentages of variation respectively (Fig. 2a). Three outliers comprising 2 in the ‘NA’ group and 1 in the ‘NPA’ group were identified and excluded from further downstream analysis. Supervised OPLS-DA models comparing children with appendicitis (NPA + PA) to NA are shown in Fig. 3a; NPA and PA groups were subsequently compared separately (Fig. 4a). The score scatterplots for each OPLS-DA analysis demonstrate separation of the two groups (Figs 3a and 4a). The variance (R^2^Y), predictivity (Q^2^), P-values, sensitivity, specificity and AUROC values of the models are demonstrated in Table 2. Twelve and thirteen serum metabolites and inflammatory mediators contributed significantly (P < 0.05) for the separation between pediatric patients with appendicitis (NPA + PA) and NA (Fig. 5a), and between NPA and PA (Fig. 6a) respectively.

**Table 2:** Summary statistics from OPLS-DA models differentiating each pair of pediatric patient groups.

	Analysis	R^2^Y:Q^2^	P-value	Sn:Sp	AUROC ± SD
Appendicitis Vs. Non-appendicitis abdominal pain	Serum metabolic and inflammatory mediator profiling	0.60:0.48	2.3E-02	0.80:0.92	0.92 ± 0.03
	Urine metabolic profiling	0.47:0.32	6.1E-07	0.74:0.85	0.85 ± 0.04
Perforated appendicitis Vs. Non-perforated appendicitis	Serum metabolic and inflammatory mediator profiling	0.66:0.37	0.001	0.60:0.97	0.88 ± 0.05
	Urine metabolic profiling	0.60:0.52	1.20E-05	0.80:1.0	0.98 ± 0.02

Abbreviations: Sn- Sensitivity, Sp- Specificity, AUROC- area under the receiver operating characteristic curve, SD-standard deviation.

### Urine metabolic profiling

Similar to serum dataset analysis, the unsupervised PCA model for the urine metabolite dataset was built based on three principal components-PC1, PC2 and PC3, contributing to 10%, 7.9% and 5% percentages of the variation respectively (Fig. 2b). A total of 8 outliers were identified, of which 6 belonged to the ‘NPA’ class and 1 to each of the other two classes; these outliers were excluded from subsequent analysis. Supervised OPLS-DA method was applied to compare the variances between each pair of patient groups. Children with appendicitis (NPA + PA) were compared with NA children (Fig. 3b) and then NPA and PA groups were separately compared (Fig. 4b). As shown in Figs 3b and 4b, the score scatterplots for each OPLS-DA analysis demonstrate separation between each of the two classes. Table 2 portrays the variance (R^2^Y), predictivity (Q^2^), sensitivity, specificity, AUROC and P-values for the supervised models. The urine metabolites that most significantly influenced the class separation in the two OPLS-DA models are illustrated in Figs 5b and 6b; in both analyses the number of metabolites identified is seven.

To ensure that the exclusion of the outliers detected in the PCA analysis did not bias the results of the supervised analyses, we recalculated the OPLS-DA models including all outliers, for both serum and urine. This had only minimal influence on the discriminative and predictive ability of the models, which are shown in the supplementary Table S2.

## Discussion

Both serum and urine analyses demonstrated promising results separating those children with abdominal pain resulting from appendicitis from other underlying etiologies. In particular, the serum and urine biomarker models identified in this study exhibited excellent specificity and AUROC values when distinguishing those with and without appendicitis as well as those with and without perforation, suggesting that the use of biomarker patterns from both biofluids may have some diagnostic potential in ruling in appendicitis. Previous studies have tried to identify individual inflammatory biomarkers or panels^12–16^ comprised of few biomarkers of acute appendicitis, but to the best of our knowledge there are currently no available studies attempting to find biomarker patterns through expansive profiling in children.

For children presenting with suspected appendicitis, prompt identification of those that need surgical or medical intervention and those that require further diagnostic investigations is crucial. Although common imaging approaches provide rapid clinically relevant information, they sometimes have risks or limitations that may confine their use. While ultrasound is a commonly used imaging method for appendicitis, it can appear equivocal in many children^17,18^ resulting in diagnostic uncertainty. Safety concerns regarding the use of computed tomography (CT) and the risk of developing radiation-induced malignancy are well documented^19^. However, despite guidelines from major professional societies warning about the risk of CT, a recent study in the United States showed that over 50% of the children with appendicitis receive a CT scan as their first diagnostic imaging^19^. Though magnetic resonance imaging (MRI) is a radiation free and efficient platform for diagnosing appendicitis, access/availability in some settings restricts its use^9^. Our biomarker profile provides an alternative diagnostic approach in which children with abdominal pain caused by appendicitis can be identified. Arguably, the current profiling process is time consuming and expensive; however, rapid advancements leading to point of care technologies are fast approaching^20–23^. As the number of metabolites and inflammatory biomarkers in the profile identified with our cohorts is relatively small, future industry partnership can lead to the development of affordable, time-sensitive and risk-free diagnostic aids to assist healthcare professionals. Future studies comparing the accuracy, resource utilization and economics between such a commercial diagnostic test and current strategies is required.

In addition to distinguishing acute appendicitis from the other conditions causing abdominal pain, triage decision-making to prioritize emergent surgical patients with suspected appendicitis requires severity stratification: detecting whether the appendix is perforated or not. Moreover, recent literature suggests that non-operative management (i.e. antibiotic therapy only) might be a reasonable strategy to treat NPA, whereas a PA requires surgical intervention to prevent any systemic infection^9^; hence optimized management strategies relies on accurate distinction between NPA and PA. However, one of the major problems in appendicitis diagnosis is the differentiation between the two stages^9^. Biomarker fingerprints derived from our pilot study could substantially distinguish children that have PA from those that have NPA, which have the potential to aid triage decisions for appendicitis management.

This study analyzed both serum and urine and found encouraging results in distinguishing the different groups of patients. However, using biomarker patterns identified from urine has significant advantages as a diagnostic aid; urine is easier and less invasive to collect than other biofluids and is collected routinely from all patients with abdominal pain to rule out common alternate diagnoses including urinary tract infection and pregnancy.

Identification of some significant metabolites and cytokines suggest that acute appendicitis might induce unique metabolic and immune processes in pediatric patients. Depleted levels of acetoacetate and acetate along with increased o-acetylcarnitine indicate higher fatty acid degradation during acute appendicitis^24–26^ compared to non-appendicitis abdominal pain (Fig. 5). Reduced concentrations of gluconate and its derivative 1,4-lactone gluconate, intermediates of phosphogluconate pathway reflect increased glycolysis in appendicitis patients^24–26^. High glucose and fatty acid breakdown in appendicitis patients suggest that energy demand increases more during appendix inflammation compared to other conditions causing abdominal pain. The presence of alanine and 4-hydroxyphenylacetate in the significant metabolite list entails alteration in the metabolism of amino acids tyrosine and phenylalanine during acute appendicitis^24–26^. High levels of 2-hydroxybutyrate and acetone, and depleted levels of citrate and eicosanoic acid in serum and urine of the children with perforated appendicitis (Fig. 6) implicate that fatty acid oxidation is even more elevated when the appendix is ruptured^24–26^.

In addition to the metabolites, several cytokines were detected to be significant in the study. The inflammatory markers C-reactive protein (CRP), IL-6, IL-10, ferritin, tissue plasminogen activator (tPA), and procalcitonin were elevated whereas the levels of cutaneous T-cell-attracting chemokine (CTACK), stem cell factor (SCF), interferon-γ-induced protein-10 (IP-10), TNF-related apoptosis-inducing ligand (TRAIL), and membrane-bound immunoglobulin (MIG) were depleted in appendicitis patients when compared with patients with other causes of abdominal pain (Fig. 5a). High levels of Hepatocyte growth factor (HGF) and IL-2, and low levels of IFN-α2, and eotaxin were exclusively detected in patients with a perforated appendix (Fig. 6a). Many of these inflammatory biomarkers are in agreement with previous studies. CRP is already in use for laboratory diagnosis of appendicitis^14,27–31^; while IL-6 and IL-10 have been found to be significant inflammatory biomarkers for acute appendicitis^13,32–35^. Similar to our finding some studies have also demonstrated that IL-6 is more elevated in advanced appendicitis compared to non-perforated simple appendicitis^35–37^. Elevated ferritin is a common indicator of inflammation^38,39^ and procalcitonin has been shown to be a reasonable indicator of the severity of acute appendicitis^40–42^. Low serum interferon in children with acute appendicitis^43^ is in line with our finding of depleted IFN-α2 level in perforated appendicitis vs. non-perforated. Overall, the metabolites and inflammatory mediators detected from pediatric patients provide distinctive biomarker patterns to distinguish between appendicitis and non-appendicitis abdominal pain, and perforated and non-perforated appendicitis.

In a previous pilot study we applied a similar biomarker profiling approach to distinguish children with appendicitis from control children without abdominal pain (manuscript submitted). There are some overlaps in the biomarkers identified in the two studies; for instance, alterations in the inflammatory mediators CRP, ferritin, TRAIL and HGF were consistent across both the studies. However, the remaining biomarkers differed, which can be expected as the study question and populations included in the two studies were different. The control cohort in the previous study included children with no abdominal pain and no signs of infection, whereas in this study the control group children had abdominal pain with suspected appendicitis. Also, the previous study did not evaluate perforated vs. non-perforated appendicitis.

There are some limitations to our study. First, the sample size for the ‘perforated appendicitis’ group (n = 14 for serum, and n = 9 for urine) is small compared to the two other groups. However, we feel that the population enrolled closely parallels the clinical setting in the PED where providers are trying to identify appendicitis from a large cohort of undifferentiated abdominal pain. Secondly, we had access to more serum samples (n = 127) than urine samples (n = 105), which precluded the integration of serum and urine metabolites. Finally, discrepancies in the volumes of some serum and urine samples caused slight inconsistencies among the total number of samples used in ^1^H NMR, GC-MS and Luminex platforms and hence we had to remove a small number of samples in the combined datasets. However, as only 6 (4.7%) serum and 3 (2.9%) urine samples were excluded, we believe, that this should not significantly affect our findings.

## Conclusion

In this study we have shown that our approach can differentiate acute appendicitis patients from other conditions with similar presentations, and stratify children based on severity of disease. The outcomes of this study suggest that the application of metabolic and inflammatory biomarker profiling in pediatric patients has the potential to improve the diagnosis of appendicitis, and in the future may aid in minimizing the current problem of significant rates of false-positive and false-negative diagnoses. Further studies on independent patient cohorts are required to validate our findings.

## Methods

### Patient enrolment

Ethics approval for this study was obtained from the Conjoint Health Research Ethics Board of the University of Calgary (Reference no. REB13-0586); all methods were performed in accordance with the relevant guidelines and regulations. All children aged 5 to 17 years presenting to the Alberta Children Hospital (ACH) PED with triage complaints of abdominal pain or vomiting were considered as potential study participants. ACH is the pediatric tertiary center for Southern Alberta, Eastern British Columbia and Western Saskatchewan with a catchment area of approximately 2 million and an annual census of over 75 thousand patient care visits. After standard initial evaluation of the child by the managing physician, the children who met the following criteria were considered eligible for enrolment: (1) suspected appendicitis, (2) an ultrasound (US) evaluation of the appendix, (3) an IV was to be or had been placed for clinical use, and (4) the child did not require PICU care directly from the PED. The children were not included if they had previous appendectomy, required active resuscitation in the PED, were pregnant, had abdominal pain for more than 5 days, had a history of illness resulting in immune suppression, were previously enrolled in the study, had an imaging study performed at a different healthcare center, or had a language barrier that interferes with informed consent. Informed consent or assent was obtained from the children and/or their caregivers prior to participation in the study.

The presence, absence, and perforation of appendicitis were confirmed by pathology examination. Children with evidence of inflamed appendix along with any presence of perforation were included in the *perforated appendicitis cohort* (PA), children with evidence of inflammation without presence of any perforation were included in the *non-perforated appendicitis cohort* (NPA), and those with appendicitis negative pathology report were included in the *non-appendicitis abdominal pain cohort* (NA). After the enrolment in the study, the clinical management of the children was left to the discretion of the treating PED and surgical teams according to our local pathway^44,45^. Blood and urine were collected from the enrolled children; detailed description on sample collection is provided in the supplementary file.

### Proton Nuclear Magnetic Resonance (^1^H NMR) Spectroscopy

A previously described protocol was followed to prepare the samples and acquire the ^1^H NMR spectra^46,47^. In brief, serum and urine samples were filtered using 3 kDa NanoSep microcentrifuge filters to remove large molecules; phosphate buffer, sodium azide and D_2_O were added to the filtrate and final pH was adjusted to 7.00 ± 0.04 for each sample. ^1^H NMR spectra were obtained from the prepared samples on a 600 MHz Bruker Ultrashield Plus NMR spectrometer (Bruker BioSpin Ltd., Milton, ON, Canada) using standard Bruker 1D spectroscopy pulse program ‘noesypr1d’^48,49^. Each spectrum was manually processed (phasing, baseline correction, referencing to the DSS peak at 0.0 ppm) and profiled using Chenomx NMR Suite 7.5 software (Chenomx Inc., Edmonton, Canada).

### Gas Chromatography-Mass Spectrometry (GC-MS)

Samples were extracted and derivatized for GC-MS following the procedure previously described^50^. Briefly, a two-phase mixture of chloroform and methanol was used to extract metabolites from serum and urine followed by separation and vacuum drying (SpeedVac, Eppendorf, Germany) of the aqueous layer. Next, metabolites were derivatized by methoxyamine hydrochloride in pyridine solution and N-methyl-N-(trimethylsilyl) trifluoroacetamide. An internal standard (phenylalanine D5) was added to each sample for obtaining the relative concentrations of the analytes. An Agilent chromatograph (Agilent Technologies Canada, Inc, Mississauga, Ontario, Canada) coupled with a Waters Mass Spectrometer (Waters Corp., Milford, MA, USA) was used to perform GC-MS. An MS range of 50–800 *m*/*z* was used for scanning and the generated mass spectra were processed using the Metabolite Detector 2.06 software (Technische Universitat Carolo-Wilhelmina zu Braunschweig, Braunschweig, Germany). Metabolites were identified based on the GOLM metabolome database^51–53^. Named metabolites are Metabolomics Standards Initiative (MSI) level 2 (putatively annotated compounds).

### Inflammatory mediator profiling

Two human cytokine and chemokine assay kits (Bio-Plex Pro Human Cytokine 21-plex Assay and Bio-Plex Pro Human Cytokine 27-plex Assay), obtained from Bio-Rad Laboratories, Inc. (Hercules, CA, USA) were used to detect the inflammatory mediators in human plasma samples; the plates were read on a Luminex 200 apparatus (Applied Cytometry Systems, Sheffield, UK). Manufacturer provided protocols were followed for sample assay. Briefly, plasma samples were thawed quickly at 37 C, centrifuged at 20800 g at 4 C for 10 minutes, and then stored on ice. Samples were diluted as recommended by the manufacturer and were distributed to appropriate wells of a 96 well flat bottom plate. The plate was then incubated at room temperature for two hours, in the dark, on a plate shaker set at 500-rotations-per-minute (rpm). 100 μL of wash buffer was used to wash wells three times with and then 25 μL of diluted biotin antibody cocktail was added. The plate was subsequently incubated, in the dark, for one-hour at room temperature on a plate shaker set at 500-rpm. Wells were again washed three times with 100 μL of wash buffer. 25 μL of diluted Streptavidin-PE then was added to each well and the plate was incubated for 30 minutes at room temperature on a plate shaker set at 500-rpm. Once again, wells were washed three times with 100 μL of wash buffer. Subsequently, 75 μL of wash buffer was added to each well. The software Bio-Plex Manager 6.0. (Bio-Rad Laboratories, Inc.) was used for the acquisition and analysis of the samples; if the coefficient of variance between two replicates was greater than 20%, the data was considered as a missing value.

### Statistical Analysis

We performed multivariate statistical analysis using SIMCA-P+ software (v12.0.1, Umetrics, Umea, Sweden)^54–58^. Common metabolites detected by both ^1^H NMR and GC-MS platforms were averaged and all the metabolites identified from the two platforms were combined, separately for serum and urine. Trending of common metabolites across platforms was ensured. Serum metabolites were then combined with the serum protein mediators. Metabolites and mediators that had >20% missing values were excluded from the statistical analysis; data preprocessing included median fold change normalization, logarithmic transformation, centering, and unit variance scaling^58^.

Unsupervised principal component analysis (PCA) was used to summarize the variation in each of the serum and urine datasets, which also showed the outliers that are situated outside of the 95% confidence interval^54^. Next, supervised orthogonal partial least squares discriminant analysis (OPLS-DA) models were developed (excluding outliers) to determine the class discriminations; the serum models were built based on the metabolites and protein mediators that had variable importance to projection (VIP) > 1; as very high numbers of metabolites were detected from urine, VIP > 1.8 was used to build the OPLS-DA models^54^. The variation (R^2^Y) and predictive ability (Q^2^) of the OPLS-DA models were calculated based on seven fold cross-validation. The coefficient of variation-analysis of variance (CV-ANOVA) p-values^59^ and the receiver operating characteristic curve (ROC) (Metz ROC Software; University of Chicago, Chicago, IL USA)^60^ were also calculated to validate the OPLS-DA models. Sensitivity and specificity were calculated from sample class prediction during sevenfold cross-validation (YpredCV) in the SIMCA-P+ software.

The OPLS-DA regression coefficients were calculated to identify the most important metabolites and protein mediators contributing to the class separations^57^; only the compounds demonstrating significant differences in concentration (P < 0.05) between the classes were considered as potential biomarkers.

As recommended in published guidelines^61^, our primary analysis (appendicitis vs no-appendicitis) met the minimal suggested number of human subjects (minimum acceptable n = 25) in each cohort.

### Data Availability

The datasets generated and analyzed during the current study are available from the corresponding authoer on reasonable request.

## Electronic supplementary material

Supplementary Information
