# Supplementary material for: The Use of Metabolomics and Inflammatory Mediator Profiling Provides a Novel Approach to Identifying Pediatric Appendicitis in the Emergency Department
Source: Sci Rep. 2018 Mar 6;8:4083. doi: 10.1038/s41598-018-22338-1 (PMC5840182; doi:10.1038/s41598-018-22338-1)
Supplement: Supplementary file 1 — Supplementary Information [file 41598_2018_22338_MOESM1_ESM.docx]

**Title:** The Use of Metabolomics and Inflammatory Mediator Profiling Provides a Novel Approach to Identifying Pediatric Appendicitis in the Emergency Department

**Authors:** Nusrat S. Shommu PhD, Craig N. Jenne PhD, Jaime Blackwood MD, Dori-Ann Martin RN BScN, Ari R. Joffe MD FRCPC, Robin Eccles MD FRCSC, Mary Brindle MD FRCSC, Ijab Khanafer MD, FRCPC, Hans J. Vogel PhD, and Graham C. Thompson MD FRCPC.

**Corresponding Author:** Graham C. Thompson; University of Calgary, Calgary, AB, Canada; Email: [graham.thompson@albertahealthservices.ca](mailto:graham.thompson@albertahealthservices.ca)

Supplementary File 1 contains:

1. Description of patient enrolment methods.
2. Sample collection procedure.
3. **Table S1:** Demographics, Clinical Features and Investigations of children with abdominal pain and suspected appendicitis based on pathology classification.
4. **Table S2:** Comparison of model strengths (R^2^Y and Q^2^ values) calculated for the OPLS-DA models with and without excluding the outliers.
5. Supplementary Reference**.**

**Patient enrolment**

Ethics approval for this study was obtained from the Conjoint Health Research Ethics Board of the University of Calgary (Reference no. REB13-0586). All children aged 5 to 17 years presenting to the Alberta Children Hospital (ACH) PED with triage complaints of abdominal pain or vomiting were considered as potential study participants. Detailed description of the inclusion and exclusion criteria is provided in the supplementary file. ACH is the pediatric tertiary center for Southern Alberta, Eastern British Columbia and Western Saskatchewan with a catchment area of approximately 2 million and an annual census of over 75 thousand patient care visits. After standard initial evaluation of the child by the managing physician, the children who met the following criteria were considered eligible for enrolment: 1) suspected appendicitis, 2) an ultrasound (US) evaluation of the appendix, 3) an IV was to be or had been placed for clinical use, and 4) the child did not require PICU care directly from the PED. The children were not included if they had previous appendectomy, required active resuscitation in the PED, were pregnant, had abdominal pain for more than 5 days, had a history of illness resulting in immune suppression, were previously enrolled in the study, had an imaging study performed at a different healthcare center, or had a language barrier that interferes with informed consent. Informed consent or assent was obtained from the children and/or their caregivers prior to participation in the study.

The presence, absence, and perforation of appendicitis were confirmed by pathology examination. Children with evidence of inflamed appendix along with any presence of perforation were included in the *perforated appendicitis cohort,* children with evidence of inflammation without presence of any perforation were included in the *non-perforated appendicitis cohort*, and those with appendicitis negative pathology report were included in the *non-appendicitis abdominal pain cohort*. After the enrolment in the study, the clinical management of the children was left to the discretion of the treating PED and surgical teams according to our local pathway[^1^](#_ENREF_1)^,^[^2^](#_ENREF_2).

**Sample collection**

Blood and urine samples were collected from the children before they received any antibiotics. For metabolic profiling, 4ml (in age 13-17yr) and 2ml (in age 5-12yr) of whole blood was collected into a 4ml serum tube containing pro-coagulant. Immediately after collection, blood samples were gently inverted several times and allowed to clot at room temperature for 60 minutes. The sample was then placed on ice for 2 hours and centrifuged at 1200g for 10 min at 4°C. The serum was then collected in a 4ml cryovial and stored at -70°C until distribution for analysis. For inflammatory mediator analysis, 4ml (in age 13-17yr) and 2ml (in age 5-12yr) of whole blood was collected into a heparin plasma tube, gently inverted several times, and immediately placed on ice. The tubes were then spun at 1200g for 10 min at 4°C in a swinging bucket centrifuge to separate plasma from the cellular fraction; plasma was then carefully transferred to a 4ml cryovial, and stored at -70°C. To rule out alternate diagnoses, urine samples are collected as part of standard evaluation in a child presenting to the PED with abdominal pain. Using sterile technique a small portion (<5mL) of urine was separated from the clinical sample for the metabolomics analysis, centrifuged at 1500g for 5 minutes in a swinging bucket centrifuge, and stored at -70°C.

**Table S1:** Demographics, Clinical Features and Investigations of children with abdominal pain and suspected appendicitis based on pathology classification.

|  | Enrolled | | Blood Profile | | Urine Profile | |
| --- | --- | --- | --- | --- | --- | --- |
|  | Appendicitis (n = 52) | No Appendicitis (n=88) | Appendicitis (n=47) | No Appendicitis (n=74) | Appendicitis (n=36) | No Appendicitis (n=66) |
| Age, mean (SD) | 11.4 (3.2) | 11.7 (3.5) | 11.4 (3.1) | 11.9 (3.5) | 11.5 (3.0) | 11.6 (3.5) |
| Male, n(%) | 30 (57.7) | 31 (35.2) | 28 (59.6) | 27 (36.5) | 19 (52.8) | 24 (36.4) |
| Prior health care visit for same illness, n(%) | 25 (48.1) | 36 (40.9) | 24 (51.1) | 32 (43.2) | 15 (41.7) | 28 (42.4) |
| Nausea, n (%) | 37 (71.2) | 58 (65.9) | 32 (68.1) | 48 (64.9) | 27 (75.0) | 44 (66.7) |
| Vomiting, n(%) | 33 (63.5) | 35 (39.8) | 29 (61.7) | 28 (37.8) | 26 (72.2) | 26 (39.4) |
| Anorexia/poor appetite, n(%) | 42 (80.8) | 59 (67.1) | 37 (78.7) | 48 (64.9) | 29 (80.6) | 44 (66.7) |
| Fever in the ED (>38C), n(%) | 19 (36.5) | 18 (20.5) | 18 (38.3) | 16 (21.6) | 13 (36.1) | 14 (21.2) |
| RLQ pain, n(%) | 38 (73.1) | 68 (77.3) | 33 (70.2) | 58 (78.4) | 25 (69.4) | 56 (84.8) |
| Pediatric Appendicitis Score, median (IQR) | 7.5 (3) | 5 (4) | 7 (3) | 5 (4) | 8 (3) | 5 (4) |
|  |  |  |  |  |  |  |
| WBC, mean (SD) | 15.7 (4.3) | 9.6 (3.9) | 15.6 (4.5) | 9.4 (3.9) | 15.9 (4.5) | 9.4 (3.9) |
| Neutrophils, mean (SD) | 13.0 (4.5) | 6.5 (3.9) | 12.8 (4.7) | 6.5 (3.8) | 13.0 (4.8) | 6.5 (3.8) |
| Ultrasound completed, n (%) | 50 (96.2) | 86 (97.7) | 45 (95.8) | 73 (98.7) | 34 (94.4) | 65 (98.5) |
| CT completed, n(%) | 3 (5.8) | 7 (8.0) | 3 (6.4) | 6 (8.1) | 3 (8.3) | 7 (10.6) |
|  |  |  |  |  |  |  |
| Surgical Consult in the ED, n(%) | 51 (98.1) | 45 (51.1) | 47 (100.0) | 34 (46.0) | 36 (100.0) | 34 (51.5) |
| ED Disposition |  |  |  |  |  |  |
| Discharged home, n(%) | 0 (0) | 66 (75.0) | 0 (0) | 57 (77.0) | 0 (0) | 48 (72.7) |
| Admit to OR, n,(%) | 50 (96.2) | 5 (5.7) | 45 (95.7) | 3 (4.1) | 34 (94.4) | 2 (3.0) |
| Admit to surgical ward for observation, n(%) | 1 (1.9) | 15 (17.0) | 1 (2.1) | 12 (16.2) | 1 (2.8) | 14 (21.2) |
| Admit to medical ward, n (%) | 1 (1.9) | 2 (2.3) | 1 (2.1) | 2 (2.7) | 1 (2.8) | 2 (3.0) |
| Appendectomy, n (%) | 52 (100.0) | 5 (5.7) | 47 (100.0) | 3 (4.1) | 36 (100.0) | 2 (3.0) |

ED – Emergency Department; RLQ – Right Lower Quadrant; WBC – White Blood Cell count; CT – Computed Tomography; MRI – Magnetic Resonance Imaging

**Table S2:** Comparison of model strengths (R^2^Y and Q^2^ values) calculated for the OPLS-DA models with and without excluding the outliers

| **Analysis** | | **R^2^Y : Q^2^**  **Outliers excluded** | **R^2^Y : Q^2^**  **Outliers included** |
| --- | --- | --- | --- |
| **Appendicitis Vs. Non-appendicitis abdominal pain** | Serum metabolic and inflammatory mediator profiling | 0.60:0.48 | 0.60:0.49 |
|  | Urine metabolic profiling | 0.47:0.32 | 0.49:0.30 |
| **Perforated appendicitis Vs. Non-perforated appendicitis** | Serum metabolic and inflammatory mediator profiling | 0.66:0.37 | 0.61:0.30 |
|  | Urine metabolic profiling | 0.60:0.52 | 0.59:0.48 |

Abbreviations: Sn- Sensitivity, Sp- Specificity, AUROC- area under the receiver operating characteristic curve, SD-standard deviation

**Supplementary Reference**

1. Weljie AM, Jirik FR. Hypoxia-induced metabolic shifts in cancer cells: moving beyond the Warburg effect. *The international journal of biochemistry & cell biology.* Jul 2011;43(7):981-989.

2. Wilcken B, Wiley V, Hammond J, Carpenter K. Screening newborns for inborn errors of metabolism by tandem mass spectrometry. *The New England journal of medicine.* Jun 5 2003;348(23):2304-2312.
